# Genetically Determined Chronic Low-Grade Inflammation and Hundreds of Health Outcomes in the UK Biobank and the FinnGen Population: A Phenome-Wide Mendelian Randomization Study

**DOI:** 10.3389/fimmu.2021.720876

**Published:** 2021-07-27

**Authors:** Shucheng Si, Jiqing Li, Marlvin Anemey Tewara, Fuzhong Xue

**Affiliations:** ^1^Department of Biostatistics, School of Public Health, Cheeloo College of Medicine, Shandong University, Jinan, China; ^2^Institute for Medical Dataology, Shandong University, Jinan, China; ^3^National Institute of Health Data Science of China, Shandong University, Jinan, China; ^4^Center for Health Promotion and Research (Former Tuberculosis Reference Laboratory), Bamenda, Cameroon

**Keywords:** phenome-wide association study (PheWAS), Mendelian randomization, C-reactive protein, inflammation, causality

## Abstract

**Background:**

C-reactive protein (CRP) has been used as a biomarker of chronic low-grade inflammation in observational studies. We aimed to determine whether genetically determined CRP was associated with hundreds of human phenotypes to guide anti-inflammatory interventions.

**Methods:**

We used individual data from the UK Biobank to perform a phenome-wide two-stage least squares (2SLS) Mendelian randomization (MR) analysis for CRP with 879 diseases. Summary-level data from the FinnGen consortium were utilized to perform phenome-wide two-sample MR analysis on 821 phenotypes. Systematic two-sample MR methods included MR-IVW, MR-WME, MR-Mod, and MR-PRESSO as sensitivity analyses combined with multivariable MR to identify robust associations. Genetic correlation analysis was applied to identify shared genetic risks.

**Results:**

We found genetically determined CRP was robustly associated with 15 diseases in the UK Biobank and 11 diseases in the FinnGen population (*P* < 0.05 for all MR analyses). CRP was positively associated with tongue cancer, bronchitis, hydronephrosis, and acute pancreatitis and negatively associated with colorectal cancer, colon cancer, cerebral ischemia, electrolyte imbalance, Parkinson’s disease, epilepsy, anemia of chronic disease, encephalitis, psychophysical visual disturbances, and aseptic necrosis of bone in the UK Biobank. There were positive associations with impetigo, vascular dementia, bipolar disorders, hypercholesterolemia, vertigo, and neurological diseases, and negative correlations with degenerative macular diseases, metatarsalgia, interstitial lung disease, and idiopathic pulmonary fibrosis, and others. in the FinnGen population. The electrolyte imbalance and anemia of chronic disease in UK Biobank and hypercholesterolemia and neurological diseases in FinnGen pass the *FDR* corrections. Neurological diseases and bipolar disorders also presented positive genetic correlations with CRP. We found no overlapping causal associations between the populations. Previous causal evidence also failed to support these associations (except for bipolar disorders).

**Conclusions:**

Genetically determined CRP was robustly associated with several diseases in the UK Biobank and the FinnGen population, but could not be replicated, suggesting heterogeneous and non-repeatable effects of CRP across populations. This implies that interventions at CRP are unlikely to result in decreased risk for most human diseases in the general population but may benefit specific high-risk populations. The limited causal evidence and potential double-sided effects remind us to be cautious about CRP interventions.

## Introduction

Inflammation plays a vital role in the development of complex human diseases (1). C-reactive protein (CRP) is a major acute-phase reactant and a sensitive biomarker of chronic low-grade inflammation (2) that has been viewed as a risk factor for cardiovascular diseases (3), cancers (2), type 2 diabetes (4), Alzheimer’s disease (AD) (5), schizophrenia (6), and autoimmune diseases (7), *etc.* Georgios et al. performed a global assessment of CRP with health-related outcomes using an umbrella review based on hundreds of observational and Mendelian randomization (MR) studies (8). Though these studies covered a wide range of outcomes, only two (cardiovascular mortality and venous thromboembolism) showed convincing evidence of association with CRP levels but were not supported by MR analysis (8). It is worth noting that when examining the MR literature for 53 outcomes of 113 observational studies, no causal association for CRP was observed for any phenotype (8). The causal effect of CRP on human health phenotypes determines its potential beneficial or adverse consequences as a target for clinical anti-inflammatory therapeutic intervention. The evidence for causal effects of CRP on multiple human phenotypes requires confirmation.

Observational studies are susceptible to unmeasured confounding factors or reverse causality (*e.g.*, higher CRP levels caused by diseases). Previous MR analyses of CRP often focused on only one or a small set of common diseases and were conducted with insufficient instrumental variables (IVs) of CRP, smaller sample sizes, summary-level datasets only, or were limited to one specific population (due to data availability), yielding inadequate study power and conflicting results. Prins et al. investigated the causal effect of CRP on 32 complex somatic and psychiatric outcomes using a large-scale cross-consortium MR analysis to provide a representative case for research in this area (9). Nevertheless, for thousands of complex human diseases, especially rare diseases for which genome-wide association study (GWAS) data are not available, even the cross-consortium MR studies may not achieve the desired results. The development of MR–phenome-wide association study (MR-PheWAS) has been proven to be helpful in these above scenarios and has been used for other important risk factors such as vitamin D (10), serum urate levels (11), and coffee consumption (12). The release of large “Biobank” datasets provides a unique opportunity and could include phenotypes that were not investigated previously due to insufficient numbers of cases. MR-PheWAS might reveal associations between an interesting exposure and nearly all disease phenotypes in a homogeneous population at once.

In the current study, we performed a systematic MR-PheWAS using individual-level data from the UK Biobank that linked to the electronic medical records (EMRs) and generated hundreds of high-throughput disease phenotypes and using summary-level data from the FinnGen population. Multiple genetic loci for CRP were taken from the latest and largest GWAS to construct genetically determined CRP (1) or IVs for subsequent two-sample MR analysis. The purpose of this study was to identify causal relationships between CRP and diseases in two cohorts and compare the differences of CRP roles across different populations and previous studies. This research could provide evidence to determine whether interventions focused on CRP may result in decreased risk of specific health-related outcomes, increased risk (or side effects), or other likely outcomes in the general population.

## Methods

### Study Population

The UK Biobank is a large-scale, detailed prospective population-based study conducted from 2006 to 2010 with over 500,000 participants aged 40–69 years (13). The cohort includes extensive phenotypic and genotypic details about its participants, including data from questionnaires, physical measures, sample assays, and genome-wide genotyping; it is linked to national medical records for longitudinal follow-up, including inpatient hospital episode records, primary care, cancer registries, and death registries (13). This study was constrained to a subset of unrelated Caucasian British individuals to minimize the influence of diverse population structures within the UK Biobank (11). We used the UK Biobank phenotype and genetic data of 384,907 participants after excluding withdrawn participants, individuals without genotypes, self-reported sex mismatched to genetic information, sex chromosome aneuploidy, >10 putative third-degree relatives in the kinship table, excessive heterozygosity (top 1%), and non-white European ancestry. Genetic quality control was performed centrally by the UK Biobank (14). For detailed information about the cohort, please refer to its official website (https://www.ukbiobank.ac.uk/).

The FinnGen study was a unique study that combined genome information with digital healthcare data of participants who were over 18 years of age and lived in Finland (15). This resource comprises prospective epidemiological cohorts, disease-based cohorts, and hospital biobank samples. Detailed information has been introduced elsewhere (16) and can be accessed on the official website (https://www.finngen.fi/fi). The summary statistics released by FinnGen includes 96,499 participants and can be acquired from the MR-Base website (http://www.mrbase.org/).

### Study Procedures

**Figure 1:**
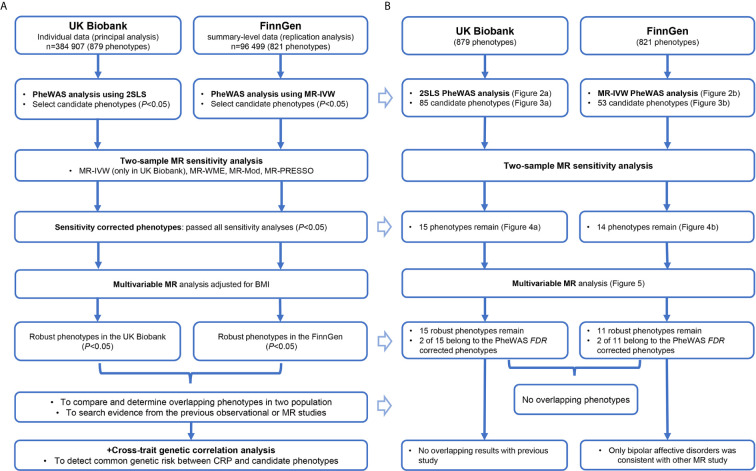
The study procedure **(A)** and summary of all results **(B)** to this research. CRP, C-reactive protein; BMI, body mass index; PheWAS, phenome-wide association study; 2SLS, two-stage least squares; MR, Mendelian randomization; *FDR*, false discovery rate, MR-IVW, inverse-variance weighted MR; MR-WME, weighted median estimator MR; MR-Mod, weighted mode-based MR; MR-PRESSO, Mendelian randomization Pleiotropy Residual Sum and Outlier.

The schematic presentation of the overall study design is shown in **Figure 1A**. First, we carried out a phenome-wide two-stage least squares (2SLS) MR analysis in the UK Biobank and inverse-variance weighted two-sample MR (MR-IVW) analysis in the FinnGen cohort to identify candidate phenotypes with significant associations. Next, we performed systematic two-sample MR as sensitivity analyses for these candidate traits to acquire sensitivity corrected phenotypes. Then, we performed a multivariable MR analysis for these phenotypes passed the sensitivity analysis to acquire the final robust associations. Finally, we compared these phenotypes [especially the phenotypes that passed the false discovery rate (*FDR*) correction in initial PheWAS] in two populations to test their consistency.

In parallel, we performed a genetic correlation analysis to identify shared genetic risks between CRP and those candidate phenotypes. The purpose of MR analysis was to determine the causal effects of CRP on diseases, while the aim of using genetic correlation analysis was to determine whether the CRP and diseases shared genetic risk. In other words, the difference between the two methods was that MR analysis explained the causal relationship of an exposure X on outcome Y; the genetic correlation analysis determined whether the traits X and Y were affected by a common genetic factor G. The use of two different methods could help explain whether the observational relationship between CRP and a specific disease was owing to causally related or shared genetic risk (genetic confounding). Details of these methods are described below.

### Genetic Instrumental Variables of CRP

For the selection of IVs, we used SNPs from the latest and largest GWAS on CRP, including >200,000 individuals of European ancestry within the “Cohorts for Heart and Aging Research in Genomic Epidemiology (CHARGE) Inflammation Working Group” (CIWG) (1). A total of 57 distinct genetic loci were selected as IVs from this summary-level dataset with the standard *P*-value threshold < 5 × 10^−8^ and *R* ^2^ <0.001. These lead variants explained up to 7.0% of the variance in circulating amounts of CRP (1), which considered that these SNPs had sufficient strength. Each variant was coded as 0, 1, and 2, and we adjusted the direction of effect values to make the effect allele corresponds to the CRP-increasing allele. The locus rs7121935 was excluded from these IVs because it has more than two alleles in the UK Biobank that could not be utilized correctly.

### Phenome Construction

We built the phenotypes using the PheCODE system, which was developed to combine one or more related International Classification of Diseases (ICD) codes into distinct disease groups that allow unbiased interrogation across multiple phenotypes in EMR-based cohorts (17). To construct the phenotypes, we applied a map designed for large biobanks to match ICD-9/10 codes to the “phecode” (18). The PheCODE system also provides a scheme to automatically exclude patients with similar or related diseases from the controls (11). We pooled both the primary and secondary ICD-9/10 codes in the UK Biobank, including hospital records, cancer registry, and death registry data, and then mapped them into the phecodes using the R package “PheWAS” (19). The phecode-mapping rules, as well as the excluding standards for each code, are available at https://phewascatalog.org/phecodes_icd10. We only included phecodes with more than 200 cases in our analysis, similar to previous studies (12). Finally, a total of 879 phenotypes were utilized in the MR-PheWAS study.

The summary statistics of disease from the FinnGen were acquired from the MR-Base platform. Among all 1,485 traits, we excluded the phenotypes with fewer than 200 cases, potential duplications, and ambiguous diseases. A total of 821 phenotypes were utilized in further studies. Because the corresponding ICD-10 codes of diseases were not released by FinnGen, we did not link them with phecodes, but rather directly adopted the given disease annotation.

### Statistical Analyses

#### One-Sample Mendelian Randomization

For individual data from the UK Biobank, the 2SLS MR method was applied to estimate the causal effects of CRP on diseases. In the first stage, the CRP was regressed on the genetic IVs using a linear regression model to generate predicted values of CRP (genetically determined CRP). In the second stage, the outcomes were regressed on the predicted CRP adjusted for age, sex, body mass index (BMI), smoking status, drinking status, and the top five genetic principal components in the multivariable logistic model. We performed a *Z*-transform for the predicted CRP to scale the causal estimators (odds ratios, ORs) corresponding to one standard deviation increment of CRP.

The “PheWAS” package was applied to test associations between genetically determined CRP and hundreds of phenotypes in the UK Biobank cohort (the second stage in 2SLS). The initial phenome-wide association analyses in the FinnGen cohort were performed using the standard MR-IVW method as a surrogate method of individual-level analysis (it is equivalent to a 2SLS analysis using individual-level data (20). Any significant signals were taken forward for further two-sample MR analyses.

#### Two-Sample Mendelian Randomization

For the candidate phenotypes detected using the phenome-wide association analyses, we performed several two-sample MR analyses in parallel to assess the robustness of causal findings. The SNP-outcome effects were acquired by performing regression of IVs on each phenotype adjusted for age, sex, and the top ten genetic principal components to control population stratification as in a previous study (21). To rule out potential reverse causality, we performed the Steiger test for each SNP using the “steiger_filtering” function and filtered out the SNPs that did not pass the directional test. The MR-IVW was taken as the primary sensitivity analysis (only for UK Biobank, since it has already been used as PheWAS method for FinnGen) with the assumption of no invalid IVs. We used the weighted median estimator (MR-WME), weighted mode-based estimate (MR-Mod), and Mendelian randomization Pleiotropy Residual Sum and Outlier (MR-PRESSO) as other sensitivity analyses. Each employs different assumptions and tolerances to horizontal pleiotropy: (1) MR-WME produces robust estimates when the proportion of invalid genetic instruments <50% (22). (2) MR-Mod is consistent when the largest number of similar causal effects estimates comes from valid IVs, even if the majority of instruments are invalid (23). (3) MR-PRESSO identifies horizontal pleiotropic outliers in a multi-instrument summary-level MR testing and returns corrected IVW estimators after removing the pleiotropic outliers (24). Here, we further used the MR-Egger to test the remaining pleiotropy after removing outliers. The MR-Egger method detects and corrects for bias due to directional pleiotropy, while the existence of horizontal pleiotropic is detected by testing whether the intercept term equals to zero (25). It was not used as one of the standards for causal estimators because of its low power, but the same direction could be viewed as additional supporting evidence. We also performed a multivariable MR (MVMR) analysis adjusted for the BMI for those associations that passed sensitivity analysis to acquire a robust result. BMI is a major determinant of CRP level (1), and obesity presented the strongest genetic correlation with CRP in our subsequent analysis, suggesting underlying pleiotropy. The phenotypes that passed sensitivity and multivariable analysis were viewed as robust associations; those that also passed the PheWAS *FDR* correction were more noteworthy.

#### Genetic Correlation Analysis

We used cross-trait Linkage Disequilibrium Score Regression (LDSC) to estimate the genetic correlation of two traits using a genome-wide dataset (26). This approach was introduced to determine whether candidate phenotypes and CRP shared genetic risks. The applied genome-wide SNPs were based on the recommended SNP list (called “w_hm3.noMHC.snplist”, including about 1.2 million SNPs based on the HapMap 3 reference panel) in the “ldsc” software, to improve computing performance and maintain common variants among different cohorts. In the UK Biobank, the effects of these SNPs on candidate phenotypes were acquired by performing genome-wide association analysis adjusted for age, sex, and top five genetic principal components. The corresponding effects of these SNPs in FinnGen were extracted from the MR-Base server using the “TwoSampleMR” package. This analysis was performed by the “ldsc” software in the Linux system using *Z*-statistics and *P*-values.

All analyses were two-tailed and performed using “ldsc” software and R software (Version 3.6.2) with R packages “TwoSampleMR”, “MRPRESSO”, and “PheWAS”.

## Results

The summary of our results corresponding to the study procedure is demonstrated in **Figure 1B**. The detail for each section follows below.

**Figure 2:**
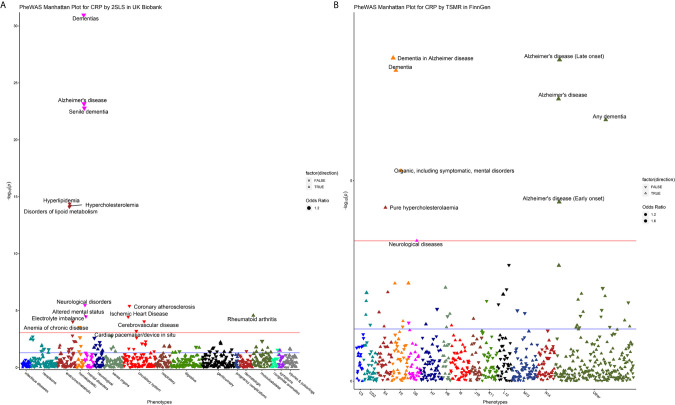
Manhattan plot for MR-PheWAS analysis in the UK Biobank **(A)** and FinnGen population **(B)**. For the Manhattan plot, the horizontal axis represents the disease group and the vertical axis represents –log_10_ (*P*) values that mean higher points indicate smaller *P*-values. The red line represents the *FDR* threshold (*q* < 0.05), and the blue line represents the experiential significant threshold (*P* < 0.05). The upward triangles indicate OR >1, while the downward triangles indicate OR <1. The size of the triangle is proportional to the OR value.

The results of MR-PheWAS scanning are presented in **Figures 2A, B**. In the UK Biobank and FinnGen, 15 and 9 diseases passed the *FDR* correction, respectively. We found several similar signals in both datasets, including AD, dementia, hypercholesterolemia, and neurological diseases. However, these overlap phenotypes across the two cohorts presented opposite effects (negative in the UK Biobank but positive in the FinnGen). Furthermore, the genetically determined CRP in the UK Biobank was negatively associated with hyperlipidemia, disorders of lipid metabolism, altered mental status, electrolyte imbalance, anemia of chronic disease, coronary atherosclerosis, ischemic heart disease, cerebrovascular disease, and cardiac pacemaker/device *in situ*, but positively associated with rheumatoid arthritis.

**Figure 3:**
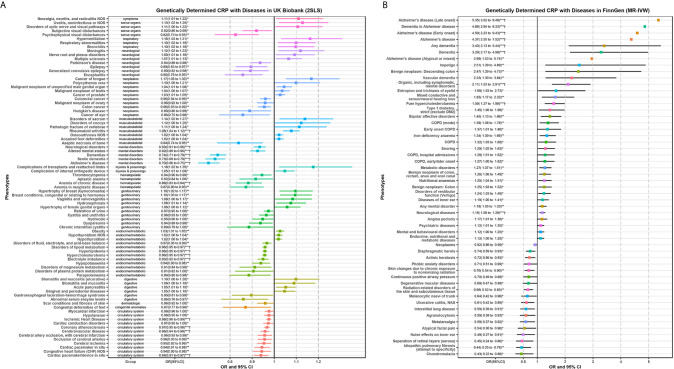
Significant phenotypes of MR-PheWAS analysis using 2SLS in the UK Biobank **(A)** and MR-IVW in the FinnGen population **(B)**. Any significant candidate phenotypes (*P* < 0.05) of MR-PheWAS for genetically determined CRP were shown in this forest plot. Several error bars only display the point because of the limitation of space. *P < 0.05; **P < 0.01; ***P < 0.001; ^†^, the phenotypes that *P-*value passed the *FDR* correction in PheWAS analysis as shown in **Figure 2**.

**Figure 3** displays the 85 candidate phenotypes that are significantly associated with genetically predicted CRP (*P* < 0.05) in the UK Biobank detected by 2SLS MR-PheWAS (**Figure 3A**) and the 53 candidate phenotypes in the FinnGen detected by MR-IVW (**Figure 3B**). The effects of genetically determined CRP showed a double-sided effect (some are positive and the others are negative) for different diseases.

**Figure 4:**
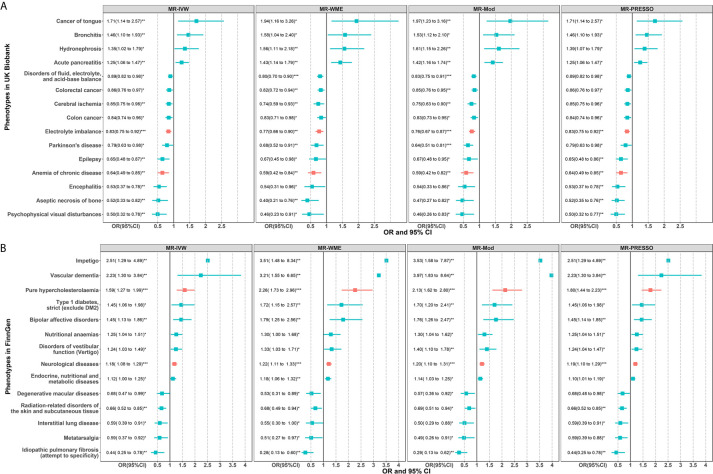
Two-sample MR sensitivity analysis for significant phenotypes of MR-PheWAS in the UK Biobank **(A)** and FinnGen population **(B)**. Only significant results in all of the four MR analyses are shown. The red error bars represent the phenotypes that also passed the *FDR* correction in the initial PheWAS analysis as shown in **Figure 2**. *P < 0.05; **P < 0.01; ***P < 0.001.

The results of these candidate phenotypes that passed sensitivity analysis are shown in **Figure 4**. A total of 15 phenotypes in the UK Biobank population satisfied the standard that was significant in all sensitivity analyses (**Figure 4A**). These sensitivity corrected associations are tongue cancer, bronchitis, hydronephrosis, acute pancreatitis, disorders of fluid, electrolyte, and acid-base balance, colorectal cancer, cerebral ischemia, colon cancer, electrolyte imbalance, Parkinson’s disease (PD), epilepsy, anemia of chronic disease, encephalitis, aseptic necrosis of bone, and psychophysical visual disturbances. The remaining 14 associations in the FinnGen population included impetigo, vascular dementia, pure hypercholesterolemia, type 1 diabetes, bipolar affective disorders, nutritional anemias, disorders of vestibular function (vertigo), neurological diseases, endocrine, nutritional and metabolic diseases, degenerative macular diseases, radiation-related disorders of the skin and subcutaneous tissue, interstitial lung disease, metatarsalgia, and idiopathic pulmonary fibrosis (**Figure 4B**).

The scatter plot for the above 15 phenotypes of UK Biobank showed that only one outlier SNP (rs12202641) was detected for the hydronephrosis but did not alter the causal estimator, and no outliers were detected for other phenotypes (**Figure S1**). MR-Egger test showed four of the 15 exposure-outcome associations remained pleiotropic (*P* for intercept term <0.05) (**Table S1**). The causal estimators of MR-Egger regression for the four pleiotropic phenotypes including disorders of fluid, electrolyte, and acid-base balance, electrolyte imbalance, PD, and cerebral ischemia still supported the results of MR sensitivity analysis (both direction and significance) (**Table S1**). The scatter plot for the 14 phenotypes in FinnGen also showed no outlier SNPs or several weak outliers that did not influence the causal estimators (**Figure S2**). The MR-Egger test showed that three of the 14 exposure-outcome associations remained pleiotropic (**Table S2**). The causal estimators of MR-Egger regression for the three pleiotropic phenotypes including pure hypercholesterolemia, vascular dementia, and bipolar affective disorders also supported the MR sensitivity analysis (**Table S2**).

**Figure 5:**
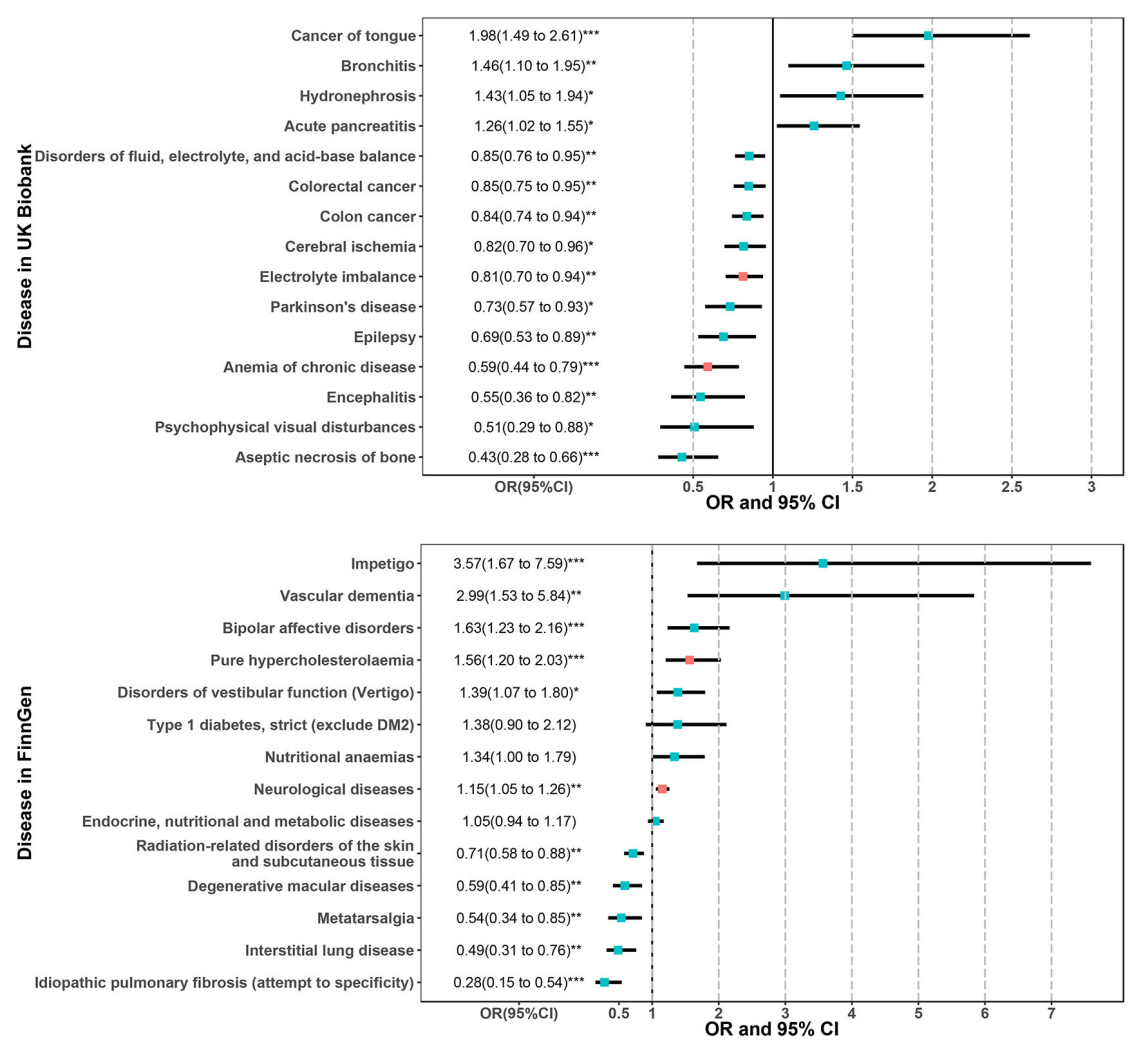
Results of multivariable MR analysis adjusted for BMI for the phenotypes passed the sensitivity analysis in the UK Biobank and FinnGen population. The red error bars represent the phenotypes that also passed the *FDR* correction in the initial PheWAS analysis as shown in **Figure 2**. *P < 0.05; **P < 0.01; ***P < 0.001.

In MVMR analysis (**Figure 5**), the 15 sensitivity corrected phenotypes in the UK Biobank remained significant after adjustment for BMI. The ORs and 95% confidence intervals (CIs) of these positive associations were 1.98 (1.49 to 2.61) for tongue cancer, 1.46 (1.10 to 1.95) for bronchitis, 1.43 (1.05 to 1.94) for hydronephrosis, and 1.26 (1.02 to 1.55) for acute pancreatitis. The negative associations were 0.85 (0.76 to 0.95) for disorders of fluid, electrolyte, and acid-base balance, 0.85 (0.75 to 0.95) for colorectal cancer, 0.84 (0.74 to 0.94) for colon cancer, 0.82 (0.70 to 0.96) for cerebral ischemia, 0.81 (0.70 to 0.94) for electrolyte imbalance, 0.73 (0.57 to 0.93) for PD, 0.69 (0.53 to 0.89) for epilepsy, 0.59 (0.44 to 0.79) for anemia of chronic disease, 0.55 (0.36 to 0.82) for encephalitis, 0.51 (0.29 to 0.88) for psychophysical visual disturbances, and 0.43 (0.28 to 0.66) for aseptic necrosis of bone. However, type 1 diabetes, nutritional anemias, and endocrine, nutritional, and metabolic diseases were no longer significant after adjusting BMI in the FinnGen population. The ORs and 95%CIs for these robust associations in the FinnGen population were 3.57 (1.67 to 7.59) for impetigo, 2.99 (1.53 to 5.84) for vascular dementia, 1.63 (1.23 to 2.16) for bipolar affective disorders, 1.56 (1.20 to 2.03) for pure hypercholesterolemia, 1.39 (1.07 to 1.80) for disorders of vestibular function (vertigo), 1.15 (1.05 to 1.26) for neurological diseases, 0.71 (0.58 to 0.88) for radiation-related disorders of the skin and subcutaneous tissue, 0.59 (0.41 to 0.85) for degenerative macular diseases, 0.54 (0.34 to 0.85) for metatarsalgia, 0.49 (0.31 to 0.76) for interstitial lung disease, and 0.28 (0.15 to 0.54) for idiopathic pulmonary fibrosis. Among the 15 and 11 robust associations in two populations, the electrolyte imbalance and anemia of chronic disease in UK Biobank and pure hypercholesterolemia and neurological diseases in FinnGen belong to the phenotypes that passed the *FDR* correction in initial PheWAS analysis. However, from hundreds of phenotypes, no overlapping phenotypes were found across the two cohorts, suggesting heterogeneity and population-specific effects of CRP levels.

It is worth noting that only four (two in UK Biobank and two in FinnGen) of the phenotypes that previously passed *FDR* correction in initial PheWAS analysis have withstood sensitivity analysis. We explored the cause using the MR-PRESSO method (to detect the outlier SNPs) and showed them in the scatter plot. For the phenotypes that passed the *FDR* correction in UK Biobank, the scatter plot (**Figure S3**) showed that a strong outlier (rs4420638) altered the effects of CRP on AD and hypercholesterolemia and others. After removing this variant and other outliers, these diseases were no longer significantly associated with CRP. For the nine phenotypes that passed the *FDR* correction in FinnGen, we found situations similar to the UK Biobank. The scatter plot (**Figure S4**) showed that the same strong outlier (rs4420638) altered the effects of CRP on these diseases except for pure hypercholesterolemia and neurological diseases. After removing the outliers, CRP was no longer associated with AD or dementia, and others. Although most of the phenotypes that passed the *FDR* threshold were due to the influence of outliers, there was actually no causal relationship. Therefore, we focused more attention on those robust phenotypes that passed multiple sensitivity tests even though their *P*-values did not reach the *FDR* threshold (of course, part of them also passed *FDR* correction).

**Figure 6:**
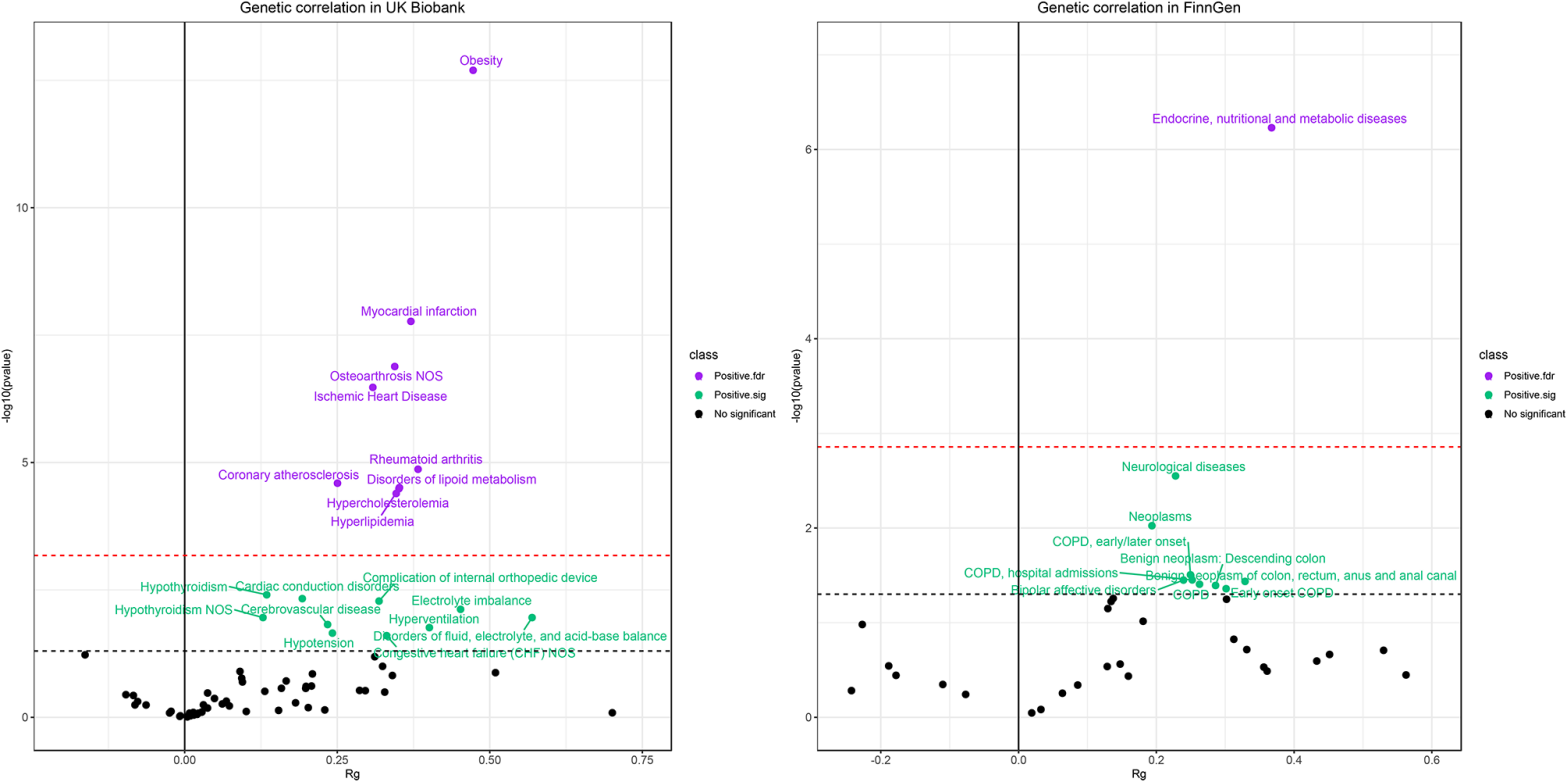
Results of genetic correlation analysis in the UK Biobank population (left) and FinnGen population (right). The red dash line represents the *FDR* threshold (*q* < 0.05) and the blue dash line represent the experiential significant threshold (*P* < 0.05). The horizontal axis represents the genetic correlation coefficients (range −1 to 1) and the vertical axis represents –log_10_(*P*) values that means higher points indicate smaller *P*-values.

**Figure 6** presents the genetic correlation among the candidate phenotypes. We found positive associations between CRP and diseases in both the UK Biobank and the FinnGen cohort, but no negative associations were observed. These findings suggest that the CRP and these diseases share common directional genetic risks. The phenotypes that passed the *FDR* correction in genetic correlation analysis were obesity, myocardial infarction, osteoarthrosis NOS, ischemic heart disease, coronary atherosclerosis, rheumatoid arthritis, hyperlipidemia, disorders of lipid metabolism, and hypercholesterolemia in the UK Biobank. Endocrine, nutritional, and metabolic diseases presented a strong genetic correlation with CRP in the FinnGen cohort. These genetic correlation coefficients ranged from 0.2 to 0.5. Traits such as cerebrovascular disease, electrolyte imbalance, hypothyroidism, congestive heart failure, neurological diseases, chronic obstructive pulmonary disease (COPD), and bipolar affective disorders also had positive genetic correlations with CRP levels (*P* < 0.05).

## Discussion

In this systematic and comprehensive phenome-wide MR analysis, we found significant associations of genetically determined CRP with 85 diseases using the 2SLS method in the UK Biobank and 53 diseases using the MR-IVW method in the FinnGen population. A total of 15 and 11 diseases were viewed as robust evidence by two-sample MR sensitivity analysis and MVMR adjustment, respectively. Among them, electrolyte imbalance and anemia of chronic disease in UK Biobank and pure hypercholesterolemia and neurological diseases in FinnGen passed the *FDR* corrections. From hundreds of phenotypes, we found no overlap disease in the two populations. Furthermore, no previous causal evidence supported these detected signals (except for the bipolar affective disorders), suggesting heterogeneous and non-repeatable effects of CRP across populations.

### Identified Associations in the UK Biobank Population

For cancer, genetically determined CRP was positively associated with tongue cancer, presenting a protective effect on colorectal and colon cancer. Graupp et al. reported that CRP was an independent prognostic marker in patients with tongue carcinoma (27). Du et al. showed that the CRP expression was associated with tongue squamous cell carcinoma tumor size, lymph node metastasis, and pathological differentiation (28). Nevertheless, it remains unclear whether the effect of CRP on the occurrence of tongue cancer is a causal relationship. The increased risk of tongue cancer may be due to the chronic inflammatory environment caused by long-term oral infection. MR analysis by Wang et al. found that CRP was not associated with an increased risk of colorectal cancer (29). The discrepancy may be attributed to the previous MR study using an older version of instrumental variables (only 19 SNPs) and being performed in a different population from ours (29). Another systematic review reported that a large proportion of studies examined CRP as a prognostic marker of cancer incidence or survival; however, MR did not highlight any evidence of causality. Therefore, further research is required to determine whether the protective effect on colorectal and colon cancer involves biological causality. Given the evidence that chronic inflammation may be linked to cancer development and progression (*e.g*., for colon cancer) (30), we do not deny the importance of inflammation. However, as described by Georgios et al., CRP is unlikely to capture the specific inflammatory mediating pathways linking inflammation to cancer development and progression (8).

For neurological disease, we found a protective role of CRP on PD and epilepsy. A systematic review and meta-analysis revealed that PD and epilepsy were associated with increased CRP levels; nevertheless, it remains questionable whether CRP serves as a causal risk factor or whether these diseases lead to a response of CRP (31, 32). In an MR study, Prins et al. found a null causal effect of CRP on PD (9). Our results from the UK Biobank were different from the causal estimator in other populations. The results for PD and epilepsy were similar to the MR study on schizophrenia, which also showed a protective effect, in contrast to an observational study (9). The hypothesis of immune response to infections in early life may partly explain such associations of CRP with decreased risk of PD and epilepsy (the amounts of acute-phase response proteins at birth are lower for individuals with non-affective psychosis than for control individuals, suggesting a weaker immune response at birth that could contribute to chronic infection in children) (1, 33). However, this hypothesis needs to be confirmed by many mechanical studies.

The 2SLS PheWAS and two-sample MR analysis found the CRP was protectively associated with disorders of fluid, electrolyte, and acid-base balance, cerebral ischemia, electrolyte imbalance, anemia of chronic disease, encephalitis, psychophysical visual disturbances, and aseptic necrosis of bone. However, no convincing observational or causal evidence supports these results. The MR-PheWAS identified the relationship between CRP and increased risk of bronchitis and acute pancreatitis. The underlying mechanism may involve the fact that both phenotypes are inflammation-related diseases; therefore, CRP might be elevated in these patients due to reverse causality.

### Identified Associations in the FinnGen Population

Genetically determined CRP was robustly associated with increased risk of vascular dementia, bipolar affective disorders, pure hypercholesterolemia, and neurological diseases. For vascular dementia, a meta-analysis demonstrated that CRP was associated with an increased risk of all-cause dementia, but not for AD (lower, insignificant hazard ratio), suggesting that the association between CRP and dementia is likely driven by other common dementia-related diseases, most notably vascular dementia (34). We also confirmed that CRP no longer influenced AD after removing outlier SNPs. Similar results for AD were replicated in the UK Biobank cohort; however, there was no causal signal for vascular dementia. For bipolar affective disorders, our findings were consistent with previous MR studies and epidemiological observations wherein elevated CRP was associated with an increased risk of bipolar disorders (9, 35, 36).

Genetically determined CRP levels also positively affected pure hypercholesterolemia though it was insignificant in the UK Biobank cohort. In an observational setting, Thongtang et al. detected a significant linkage between CRP metabolism and triglyceride-rich apo B-100 and apo B-48 catabolism using stable isotope methodology; however, there was no further experimental evidence to validate this finding (37). Our genetic correlation suggested a consistent direction of CRP with hypercholesterolemia and hyperlipidemia. This finding suggested that the CRP and hypercholesterolemia shared genetic risk factors and the relationship between them may be due in part to the genetic pleiotropy. The causal effect of CRP on neurological diseases may also be attributed to the shared genetic risk as the genetic correlation showed. The other positive associations with impetigo and disorders of vestibular function may be linked to the effects of inflammation that required more research for validation.

Genetically determined CRP displayed negative associations with degenerative macular diseases, metatarsalgia, interstitial lung disease, idiopathic pulmonary fibrosis, and radiation-related disorders of the skin and subcutaneous tissue. The results of degenerative macular diseases were different from the MR study of CRP on age-related macular degeneration (AMD) that highlighted higher circulating CRP levels leading to increases in risk for all forms of AMD (38). An observational study showed that baseline CRP levels predicted long-term interstitial lung disease progression, and another found that high-positive CRP was associated with rheumatoid arthritis-associated interstitial lung disease, which also conflicts with our causal evidence (39, 40). The observational evidence for the effect of CRP on metatarsalgia, idiopathic pulmonary fibrosis, and disorders of the skin remains lacking. Therefore, the exact mechanism of these relationships requires a well-designed experimental analysis.

### Genetic Correlation of CRP With Phenotypes

Unlike the MR analysis, we found consistent positive associations of CRP with other phenotypes. However, genetic correlation does not represent causality, while it reflects shared genetic risks and may indicate potential genetic pleiotropy. For example, we found extensive genetic correlations of cardiovascular disease such as ischemic heart disease and coronary atherosclerosis, but no causal associations were detected by MR analysis. Other studies reported that CRP was associated with coronary artery disease, suggesting a beneficial effect of lowering inflammation as well (1, 41). Such observational associations may be driven by common genetic risk factors that could be genetic confounders but not causal actors.

We also found the most potent genetic correlation between CRP and obesity in the UK Biobank and Endocrine, nutritional and metabolic diseases in the FinnGen cohort (obesity belongs to the endocrine, nutritional and metabolic diseases according to the ICD-10 tree). Considering that obesity is one of the main determinants of chronic low-grade inflammation in the general population, the relationship between CRP and many phenotypes may be confounded by the effects of obesity.

### Interpretation and Application of Key Findings

In summary, our findings are consistent with previous MR studies that reported no associations of genetically determined CRP levels with inflammation-related outcomes including coronary heart disease (42), type 2 diabetes (43), and AD (44). Genetically determined CRP was significantly associated with several phenotypes in the UK Biobank and the FinnGen population. Most of the associations that passed *FDR* correction (*e.g.*, AD) were affected by outlier SNPs and showed null causality, whereas, those remaining significant phenotypes (*i.e.*, that passed sensitivity analysis) were not repeatable across two populations.

We found some inconsistent associations in UK Biobank and FinnGen, although they passed the *FDR* correction, including Alzheimer’s disease, dementia, hypercholesterolemia, and neurological diseases. On sensitivity analysis, we identified a strong outlier (rs4420638) that altered the effects of CRP on these diseases. This strong locus showed an opposite SNP-outcome effect in different datasets. As reported in another study, rs4420638 is a non-coding variant in the *APOE* region near the *APOC1* gene; the association between it and AD is not always robust because the association between rs4420638 and AD was eliminated by *APOE* adjustment (45). Therefore, this locus may not be an effective instrumental variable for CRP, and it is even more likely to be relevant to the outcome (especially lipid-related and neurological diseases) rather than the exposure. Therefore, the effects of genetically determined CRP on these unexpected diseases in the UK Biobank and the FinnGen were most likely due to this outlier, and most likely, there is no causality. It is necessary to explore further these associations based on the biological role of this locus in the future.

We concluded that genetically determined CRP has double-sided effects for these robust associations in the two populations. This finding means that the use of clinical anti-inflammatory intervention measures against CRP would potentially benefit the prevention of some diseases such as tongue cancer, bronchitis, hydronephrosis, acute pancreatitis, and bipolar affective disorders but may yield potentially increased risk for some diseases such as colorectal cancer, colon cancer, cerebral ischemia, electrolyte imbalance, PD, encephalitis, epilepsy, degenerative macular diseases, and interstitial lung disease. Similar to the double-sided effects in the study of Jung et al., genetically determined CRP exhibited its effects on the decreased colorectal cancer risk in non-viscerally obese and high-fat diet subgroups but was associated with an increased risk for other individuals (46). This finding means that the interventions in clinical practice may not be suitable for the general population but may be applied to specific high-risk individuals for specific diseases. For example, the electrolyte imbalance, anemia of chronic disease, hypercholesterolemia, and neurological diseases are the most noteworthy phenotypes in our research because they passed both sensitivity analysis and *FDR* correction. Measures could be taken to keep an ideal CRP level to avoid the electrolyte imbalance and anemia of chronic disease, while effective anti-CRP intervention may benefit the prevention of hypercholesterolemia and neurological diseases. In addition, because its effects in bipolar disorder have been validated in studies, reasonable CRP control may also be beneficial. Moreover, the generalizability of the anti-inflammatory intervention measures in clinical practice for other populations would require further investigation because our findings suggest a population heterogeneity of CRP roles. Therefore, we should be cautious about CRP interventions.

In the context of the critical role of inflammation on widespread diseases, we still cannot rule out the potential pathogenic effects of inflammation even though the effects of CRP are controversial. Although CRP functions as a biomarker for inflammation, it does not represent the inflammation itself. This means the pathogenic effect of inflammation is unlikely to be attributable to CRP. Future research should focus on other inflammation-related factors such as the interleukin family, inflammatory cytokines, and white blood cell traits. Furthermore, because our research was performed in a high-throughput setting, the detected signals may also be partly due to the chance, because most of the robust findings did not pass the multiple corrections. Combined with previous observational studies, MR studies, systematic reviews, and our two-cohort MR-PheWAS analysis, these conflicting results suggest that the effects of CRP may be heterogeneous across the population. Finally, given our suggestive results showing the double-sided effects of CRP, CRP-based anti-inflammatory interventions may also cause potential risks or side effects for many diseases.

### Strengths and Limitations

A strength of this study was that we utilized the latest and largest IV sets for CRP that captured the maximum variance in the current background. The utilization of the entire UK Biobank individual dataset provided an unprecedented opportunity to assess hundreds of high-throughput phenotypes by using phenome-wide 2SLS analysis and two-sample MR analysis in parallel to detect any possible causal evidence in one homogeneous population. Then, we conducted MR analysis for hundreds of phenotypes using summary-level data from the FinnGen cohort to explore the reproducible causality. Under the comprehensive MR framework, we used four classic methods to avoid false positives due to the limitations of MR methods and the large sample size. Genetic correlation helps us to explain the diverse relationships between CRP and phenotypes from the view of shared genetic risk. Of course, some limitations should be pointed out. First, genetically determined CRP measures lifelong CRP level and only acquires population-averaged effects rather than short-term responses to inflammation. Second, our study only included participants of European ancestry, and the results may not be generalizable to other races or ethnicities. Third, the genetic correlation showed extensive genetic risks between the CRP and some diseases, though some MR methods might avoid potential pleiotropy; nevertheless, these methods could not completely control genetic confounders.

## Conclusion

Genetically determined CRP levels were associated with many diseases in the UK Biobank and the FinnGen population; however, only a few could be viewed as robust associations. These phenotypes could not be replicated across two populations and failed to be verified by previous observational or MR studies. Therefore, these findings have limited evidence to support the notion of the causal role of CRP on human diseases. This finding suggests that interventions for CRP are unlikely to result in decreased risk for the majority of human health-related outcomes. The limited causal evidence and potential double-sided effects remind us to be cautious about CRP interventions.

## Data Availability Statement

Publicly available datasets were analyzed in this study. This data can be found here: Researchers may have access to the UK Biobank dataset by submitting an application to the UK Biobank official website (https://www.ukbiobank.ac.uk/). This study used the UK Biobank resource with the application ID: 51470. The summary-level data from the FinnGen cohort could be download from the MR-Base platform (https://www.mrbase.org/).

## Author Contributions

SS and FX conceived and designed the study. SS did the statistical analyses and drafted the initial manuscript. FX, SS, and JL revised the manuscript critically for important intellectual content. MAT revised the English language. All authors contributed to the article and approved the submitted version.

## Funding

This research was funded by the National Natural Science Foundation of China (81773547), the National Key Research and Development Program (2020YFC2003500), and the Natural Science Foundation of Shandong Province (ZR2019ZD02). The corresponding author FX obtained the funding. The funders had no role in this work.

## Conflict of Interest

The authors declare that the research was conducted in the absence of any commercial or financial relationships that could be construed as a potential conflict of interest.

## Publisher’s Note

All claims expressed in this article are solely those of the authors and do not necessarily represent those of their affiliated organizations, or those of the publisher, the editors and the reviewers. Any product that may be evaluated in this article, or claim that may be made by its manufacturer, is not guaranteed or endorsed by the publisher.

## Supplementary Material

Supplementary Table 1 MR-Egger analysis for the remaining pleiotropy and causal estimators of 15 phenotypes passed the sensitivity analysis in the UK Biobank population.

Supplementary Table 2 MR-Egger analysis for the remaining pleiotropy and causal estimators of 14 phenotypes passed the sensitivity analysis in the FinnGen population.

Supplementary Figure 1 The scatter plot for the 15 phenotypes passed MR sensitivity analyses in the UK Biobank population. The outliers were detected by the MR-PRESSO method. Each regression line represents a two-sample method, and the slope represents the causal estimator.

Supplementary Figure 2 The scatter plot for the 14 phenotypes passed MR sensitivity analyses in the FinnGen population. The outliers were detected by the MR-PRESSO method. Each regression line represents a two-sample method, and the slope represents the causal estimator.

Supplementary Figure 3 The scatter plot for the 15 phenotypes that passed the *FDR* correction in MR-PheWAS scanning in the UK Biobank population. The outliers were detected by the MR-PRESSO method. Each regression line represents a two-sample method, and the slope represents the causal estimator.

Supplementary Figure 4 The scatter plot for the nine phenotypes that passed the *FDR* correction in MR-PheWAS scanning in the FinnGen population. The outliers were detected by the MR-PRESSO method. Each regression line represents a two-sample method, and the slope represents the causal estimator.

## Abbreviations

CRP, C-reactive protein; 2SLS, two-stage least squares; AD, Alzheimer’s disease; MR, Mendelian randomization; IVs, instrumental variables; MR-PheWAS, MR–Phenome Wide Association Study; EMRs, electronic medical records; GRS, genetic risk score; ORs, odds ratios; CIs, confidence intervals; ICD, international classification of diseases; BMI, body mass index; MR-IVW, inverse-variance weighted MR; MR-WME, weighted median estimator MR; MR-Mod, weighted Mode-based MR; MR-PRESSO, Mendelian randomization Pleiotropy Residual Sum and Outlier; LDSC, Linkage Disequilibrium Score Regression; COPD, chronic obstructive pulmonary disease; PD, Parkinson’s disease.
