# Supplementary figures and images for: Genetically Determined Chronic Low-Grade Inflammation and Hundreds of Health Outcomes in the UK Biobank and the FinnGen Population: A Phenome-Wide Mendelian Randomization Study
Source: Front Immunol. 2021 Jul 27;12:720876. doi: 10.3389/fimmu.2021.720876 (PMC8353321; doi:10.3389/fimmu.2021.720876)

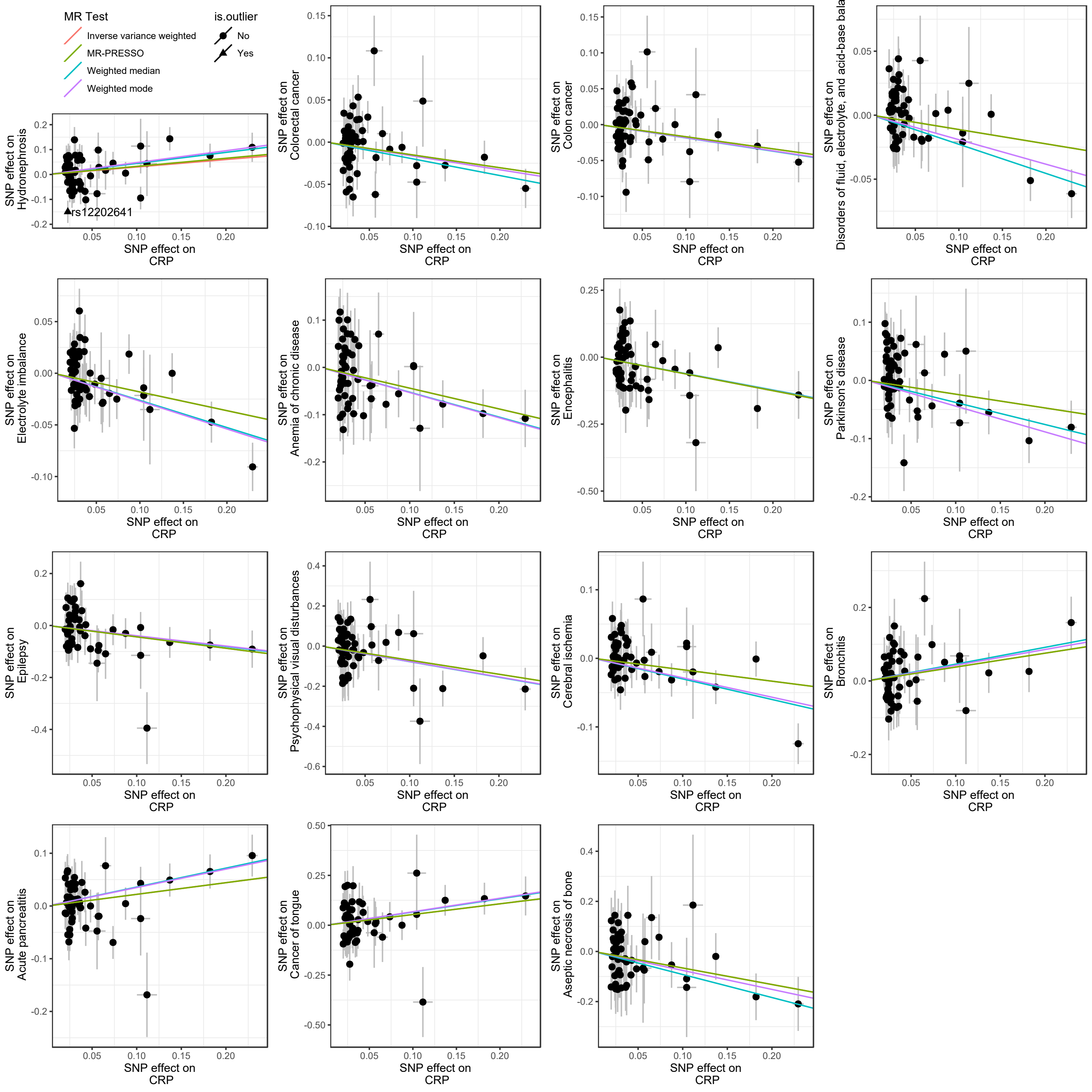

Supplement: Supplementary Figure 1 — The scatter plot for the 15 phenotypes passed MR sensitivity analyses in the UK Biobank population. The outliers were detected by the MR-PRESSO method. Each regression line represents a two-sample method, and the slope represents the causal estimator. [file DataSheet_1.pdf]

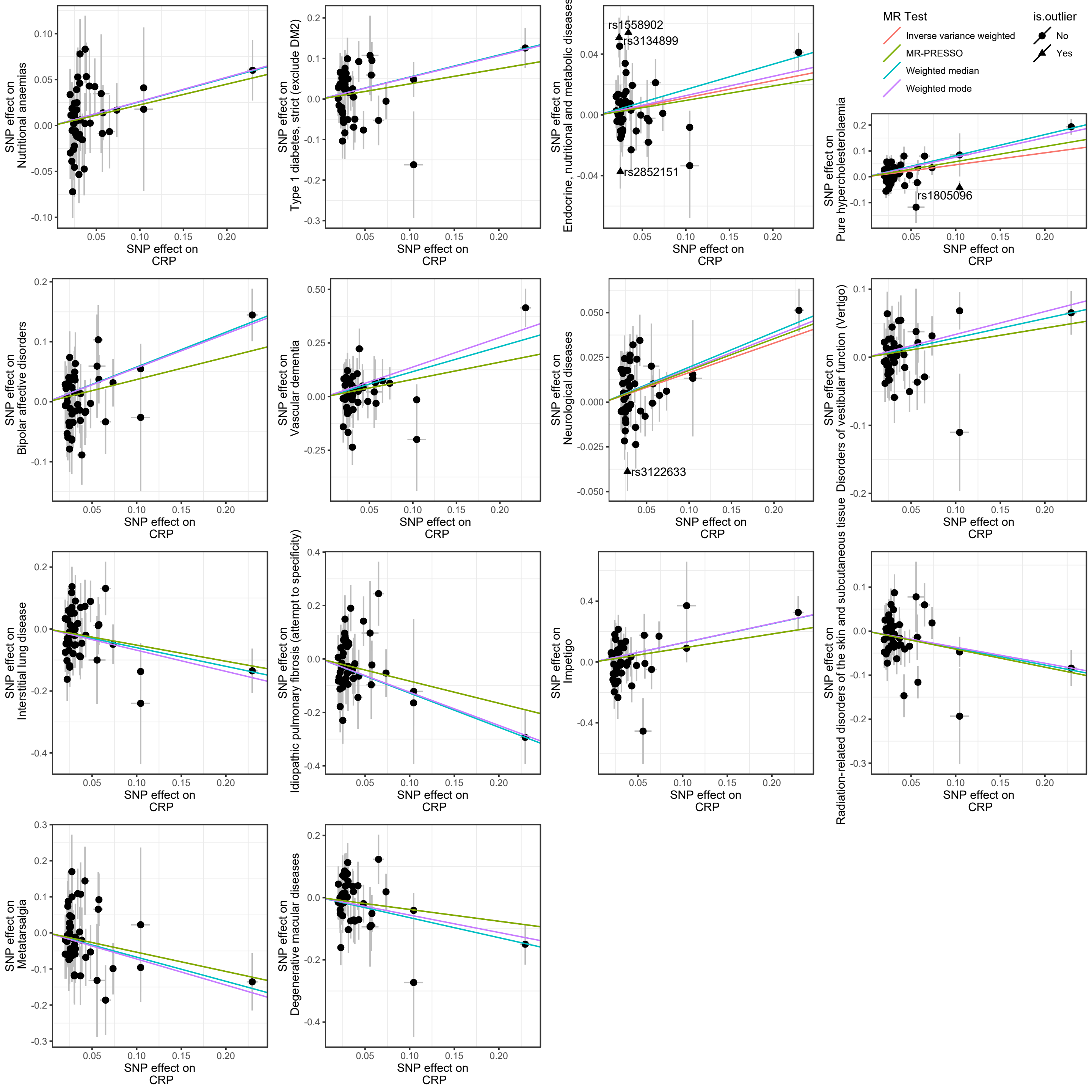

Supplement: Supplementary Figure 2 — The scatter plot for the 14 phenotypes passed MR sensitivity analyses in the FinnGen population. The outliers were detected by the MR-PRESSO method. Each regression line represents a two-sample method, and the slope represents the causal estimator. [file DataSheet_2.pdf]

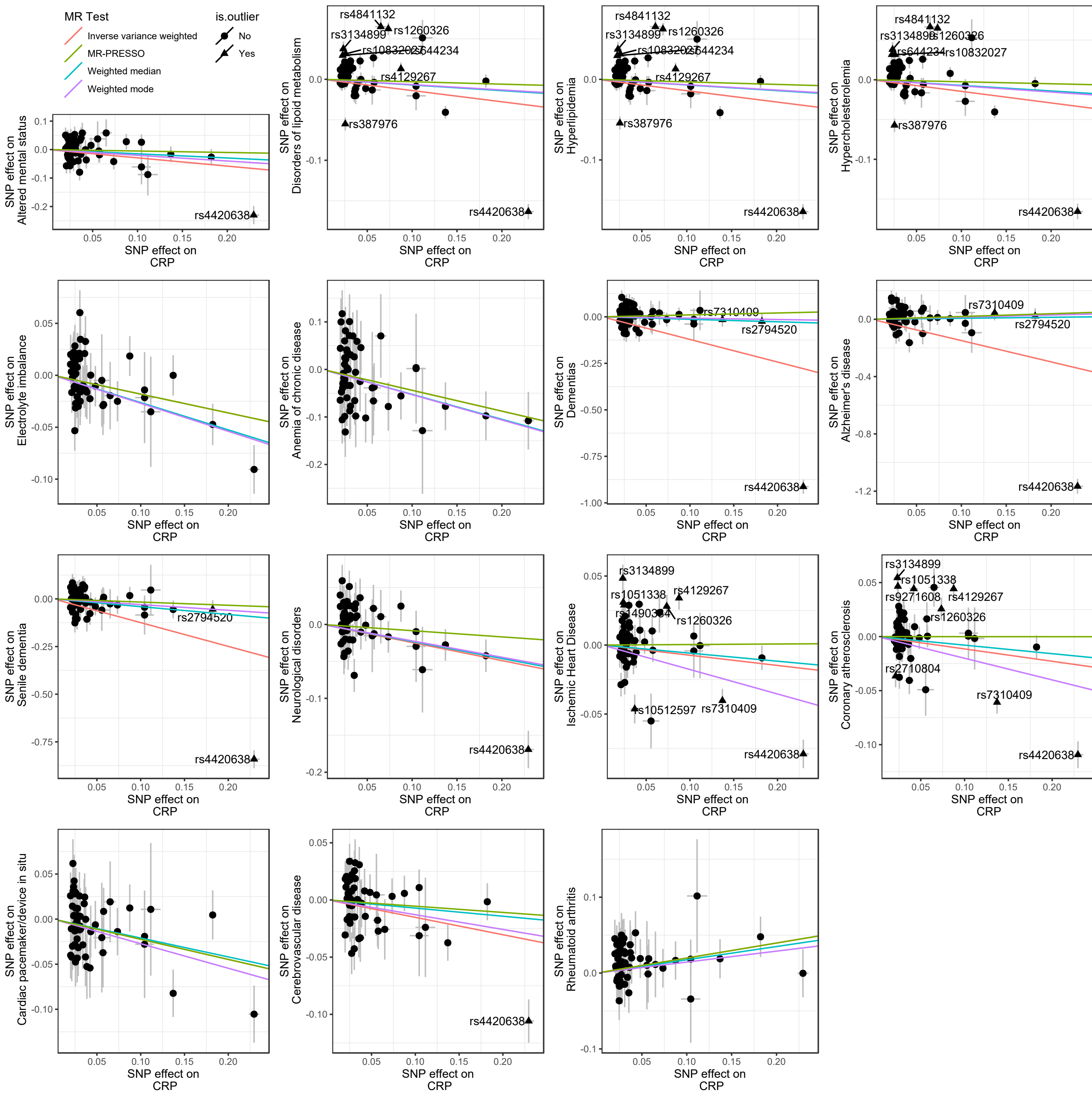

Supplement: Supplementary Figure 3 — The scatter plot for the 15 phenotypes that passed the FDR correction in MR-PheWAS scanning in the UK Biobank population. The outliers were detected by the MR-PRESSO method. Each regression line represents a two-sample method, and the slope represents the causal estimator. [file DataSheet_3.pdf]

MR Test

- Inverse variance weighted
- MR-PRESSO
- Weighted median
- Weighted mode

is.outlier

- No
- Yes

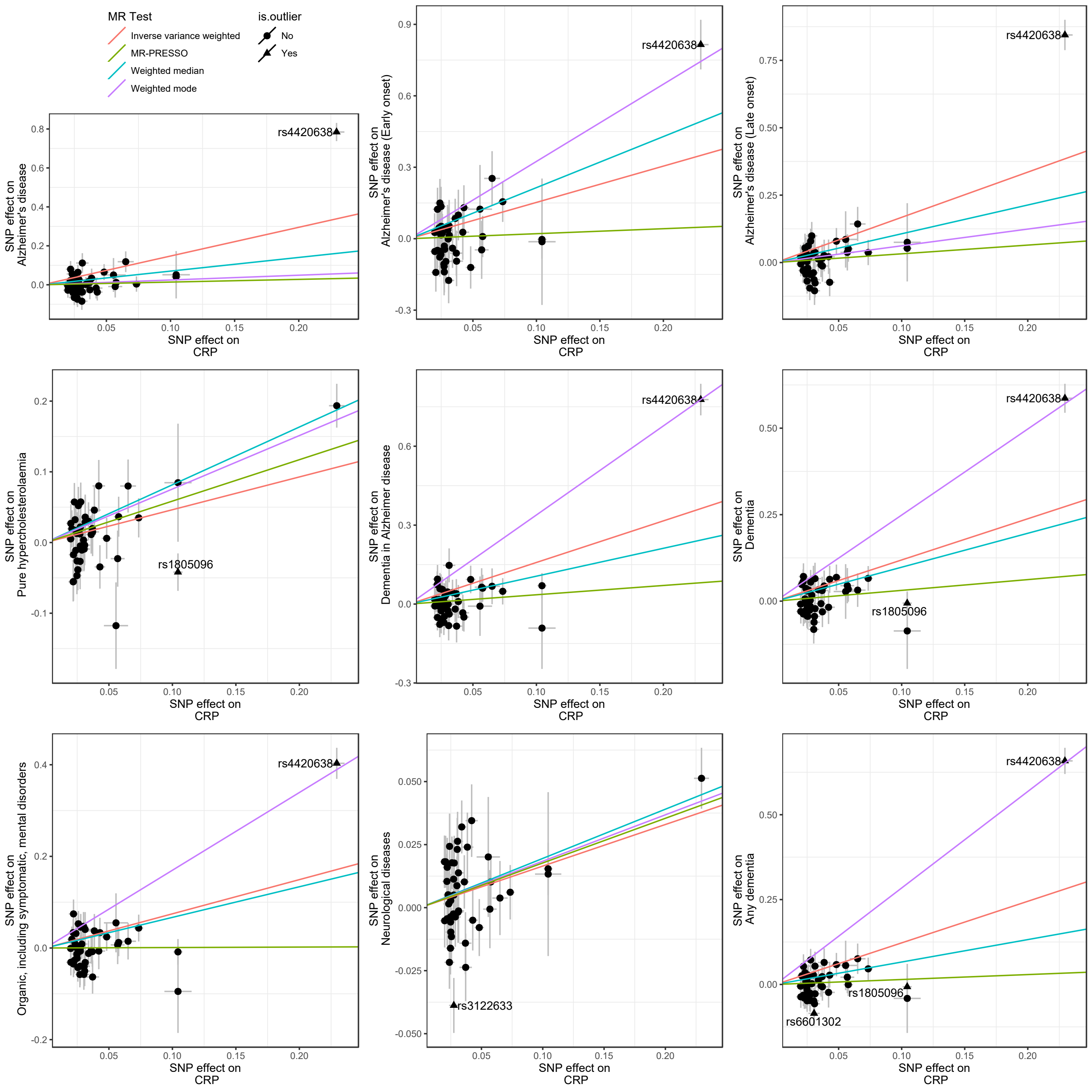

Supplement: Supplementary Figure 4 — The scatter plot for the nine phenotypes that passed the FDR correction in MR-PheWAS scanning in the FinnGen population. The outliers were detected by the MR-PRESSO method. Each regression line represents a two-sample method, and the slope represents the causal estimator. [file DataSheet_4.pdf]
